# Coronary artery lumen volume index as a marker of flow-limiting atherosclerosis—validation against ^13^N-ammonia positron emission tomography

**DOI:** 10.1007/s00330-020-07586-y

**Published:** 2021-01-16

**Authors:** Georgios Benetos, Dominik C. Benz, Georgios P. Rampidis, Andreas A. Giannopoulos, Elia von Felten, Adam Bakula, Aleksandra Sustar, Tobias A. Fuchs, Aju P. Pazhenkottil, Catherine Gebhard, Philipp A. Kaufmann, Christoph Gräni, Ronny R. Buechel

**Affiliations:** 1 Department of Nuclear Medicine, University Hospital and University Zurich, Raemistrasse 100, 8091 Zurich, Switzerland; 2 Department of Cardiology, Inselspital, Bern University Hospital, University of Bern, Bern, Switzerland

**Keywords:** Computed tomography angiography, Positron emission tomography, Myocardial ischemia

## Abstract

**Objectives:**

Coronary artery volume indexed to left myocardial mass (CAVi), derived from coronary computed tomography angiography (CCTA), has been proposed as an indicator of diffuse atherosclerosis. We investigated the association of CAVi with quantitative flow parameters and its ability to predict ischemia as derived from ^13^N-ammonia positron emission tomography myocardial perfusion imaging (PET-MPI).

**Methods:**

Sixty patients who underwent hybrid CCTA/PET-MPI due to suspected CAD were retrospectively included. CAVi was defined as total coronary artery lumen volume over myocardial mass, both derived from CCTA. From PET-MPI, quantitative stress and rest myocardial blood flow (MBF) and myocardial flow reserve (MFR) were obtained and correlated with CAVi, and semi-quantitative perfusion images were analyzed for the presence of ischemia. Harrell’s c-statistic and net reclassification improvement (NRI) analysis were performed to evaluate the incremental value of CAVi over the CCTA model (i.e., stenosis > 50% and > 70%).

**Results:**

CAVi correlated moderately with stress MBF and MFR (*R* = 0.50, *p* < 0.001, and *R* = 0.39, *p* = 0.002). Mean stress MBF and MFR were lower in patients with low (i.e., ≤ 20.2 mm^3^/g, *n* = 24) versus high (i.e., > 20.2 mm^3^/g, *n* = 36) CAVi (*p* < 0.001 for both comparisons). CAVi was independently associated with abnormal stress MBF (OR 0.90, 95% CI 0.82–0.998, *p* = 0.045). CAVi increased the predictive ability of the CCTA model for abnormal stress MBF and ischemia (c-statistic 0.763 versus 0.596, *p*_diff_ < 0.05 and 0.770 versus 0.645, *p*_diff_ < 0.05, NRI 0.84, *p* = 0.001 and 0.96, *p* < 0.001, respectively).

**Conclusions:**

CAVi exhibits incremental value to predict both abnormal stress MBF and ischemia over CCTA alone.

**Key Points:**

• *Coronary artery volume indexed to left myocardial mass (CAVi), derived from coronary computed tomography angiography (CCTA), is correlated with myocardial blood flow indices derived from* ^*13*^*N-ammonia positron emission tomography myocardial perfusion imaging.*

*• CAVi is independently associated with abnormal stress myocardial blood flow.*

*• CAVi provides incremental diagnostic value over CCTA for both abnormal stress MBF and ischemia.*

## Introduction

Coronary computed tomography angiography (CCTA) is a valuable tool for the non-invasive anatomic assessment of coronary atherosclerosis due to its excellent negative predictive value. A limitation of CCTA is its moderate positive predictive value [1, 2].

Consequently, there is growing interest in parameters derived from anatomy, which exhibit the ability to assess the functional relevance of coronary stenosis. Among them, coronary artery lumen volume indexed to left ventricular mass (CAVi) has been recently proposed as a novel CCTA-derived marker for non-obstructive but flow-limiting coronary atherosclerosis with prognostic value [3]. Furthermore, CAVi has been associated with an abnormal fractional flow reserve (FFR), as derived from invasive coronary angiography [4]. Aside from focal coronary artery stenosis, diffuse atherosclerosis in epicardial vessels constitutes an additional mechanism affecting myocardial blood flow (MBF) [5, 6]. As CAVi is not solely dependent on focal stenosis but rather reflects changes in coronary artery lumen among the entire coronary tree, it may be a potential surrogate marker for changes in global and/or regional MBF.

Positron emission tomography myocardial perfusion imaging (PET-MPI) is considered the gold standard for the non-invasive quantitative assessment of MBF (in milliliters per gram per minute) with independent prognostic value [7]. Potential association between CAVi and quantitative PET-derived parameters would further corroborate the validity of a concept pertaining the flow-limiting effects of diffuse atherosclerosis.

In the present study, we aimed to investigate the association of CAVi with quantitative flow parameters and its ability to predict ischemia as derived from ^13^N-ammonia PET-MPI.

## Materials and methods

### Study population

We retrospectively identified patients who underwent both CCTA and PET-MPI within a maximum interval of 90 days at our institution due to suspected coronary artery disease (CAD). Patients with a history of previous percutaneous coronary intervention (PCI) or coronary artery bypass grafting (CABG), inadequate CT image quality, resulting in ≥ 1 non-evaluable coronary segments, or incomplete segmentation of the left ventricular mass, were excluded.

Baseline demographic and clinical characteristics and traditional cardiovascular risk factors were recorded in all patients. The study protocol was approved by the local ethics committee (BASEC-Nr. 2016-00177 and 2018-00508), and the need for written informed consent was waived.

### CCTA data acquisition and assessment

All patients were pre-treated with 2.5 mg sublingual spray of isosorbide dinitrate and, if necessary, with up to 30 mg intravenous metoprolol to achieve a heart rate < 65 bpm.

Contrast-enhanced CCTA was performed either on 64-slice (*n* = 20; LightSpeed VCT or Discovery HD 750, both GE Healthcare) or a 256-slice scanner (*n* = 40, CT Revolution, GE Healthcare) CT scanner, using axial scanning with prospective ECG triggering, as previously described [8, 9]. In brief, collimation was 64 × 0.625 mm with an axial resolution of 0.23 × 0.23 mm, z-coverage 40 mm with an increment of 35 mm and gantry rotation time 350 ms for the 64-slice scanner, collimation 256 × 0.625 mm with an axial resolution of 0.23 × 0.23 mm, z-coverage 160 mm and gantry rotation time 280 ms for the 256-slice scanner. The smallest possible window at one distinct mid-diastolic phase of the RR cycle (i.e., setting the ECG trigger at 75%) was chosen.

All vessels were assessed for the presence of atherosclerotic plaques. Separate analyses were performed for coronary stenosis > 50% and > 70% [10].

### Assessment of CAVi

The coronary arterial tree was segmented and extracted from the imaging data and analyzed using dedicated software (CardIQ Xpress-Auto Coronary Analysis, GE Healthcare). The three main arteries (left main stem and left anterior descending artery, left circumflex, and right coronary artery) and their branches were segmented distally up to a minimal vessel diameter of 1.5 mm, in order to ensure adequate distal contrast opacification and subsequent reliable volume calculations. The software automatically identified and contoured the lumen and different plaque components based on scan-specific attenuation thresholds in each individual scan [11]. Automatic contouring was manually corrected where required. The coronary artery volume (i.e., lumen) was then calculated in mm^3^. The software tool has already been validated versus intravascular ultrasound [12]. For the left ventricular (LV) mass measurements, endocardial and epicardial contours were automatically drawn and manually adjusted where needed. LV mass was calculated in gram using dedicated software (CardIQ Function Xpress, GE Healthcare). Lastly, indexed coronary volume for each patient was calculated by dividing the total coronary volume by the LV mass, resulting in the CAVi. The measurement can be acquired in 10 min per patient on average (range 5 to 15 min) and the methodology yields excellent intra- and interreader agreement (0.99 and 0.98, both *p* < 0.001, respectively).[3]

### ^13^N-ammonia PET imaging and data analysis

PET acquisition and analysis protocol have been previously described [13, 14]. In brief, all patients underwent ^13^N-ammonia at rest and during adenosine-induced stress at a standard rate (0.14 mg/min/kg). Images were acquired either on a Discovery (LS/RX) PET/CT scanner or on an Advance PET scanner (both GE Healthcare).

Quantitative global MBF and myocardial flow reserve (MFR) were calculated using commercially available software (PMOD version 3.7; PMOD Technologies Ltd.). In brief, a volume of interest was placed in the blood-pool of the left and the right ventricle. Consequently, myocardial and blood-pool time-activity curves (TAC) were obtained from dynamic frames, corrected for radioisotope decay. Stress and rest MBF values were estimated by model fitting of the blood-pool and myocardial TACs in stress and rest, respectively. The left ventricular wall was divided into 17 segments [13, 15, 16]. MFR was accordingly calculated for each segment as the ratio of stress to rest MBF [13, 14]. An MFR < 2 or a stress MBF < 1.79 ml/g/min were considered abnormal [16, 17]. Lastly, regional MBF and MFR values of the 17 segments were recorded for each coronary territory based on standard distribution models [15].

Semi-quantitative segmental normalized relative ^13^N-ammonia uptake was recorded according to the guidelines [18]. Presence of ischemia was defined as a summed segmental difference score (SDS) ≥ 2 [18].

### Statistical analysis

Continuous variables were expressed as mean ± standard deviation while categorical variables as absolute numbers and frequencies. Comparison of continuous variables was performed using either Student’s *t* test or Mann-Whitney *U* test as appropriate. Assessment for normality of data distribution was evaluated by the Kolmogorov-Smirnov test. The Pearson’s correlation coefficient was used to assess the correlation between CAVi and quantitative PET parameters. A two-tailed *p* value < 0.05 was considered statistically significant. A receiver operator characteristics (ROC) curve analysis was performed to investigate the performance of CAVi in predicting ischemia, and the area under the curve (AUC) was calculated. Consequently, Youden’s index was used to calculate the optimal cutoff for CAVi. Moreover, multivariate logistic regression analysis was used to determine whether there is an independent association of CAVi with stress MBF and ischemia.

To further investigate the incremental predictive value of CAVi in predicting abnormal stress MBF or ischemia, the following models were selected and compared using Harrell’s c-statistics: Model (1): stenosis > 50%; model (2): stenosis > 70%; model (3): CAVi; model (4): stenosis > 50% + CAVi; model (5): stenosis > 70% + CAVi. Additionally, we explored the impact of the addition of CAVi as a continuous variable on a basic model based on CCTA stenosis severity (i.e., > 50% or > 70%) on integrated discrimination improvement (IDI) and net reclassification improvement index (NRI). Statistical analysis was performed using SPSS software (version 25, IBM), while MedCalc software (MedCalc Software Ltd.) was used for the comparison of AUCs.

## Results

### Study population

**Fig. 1:**
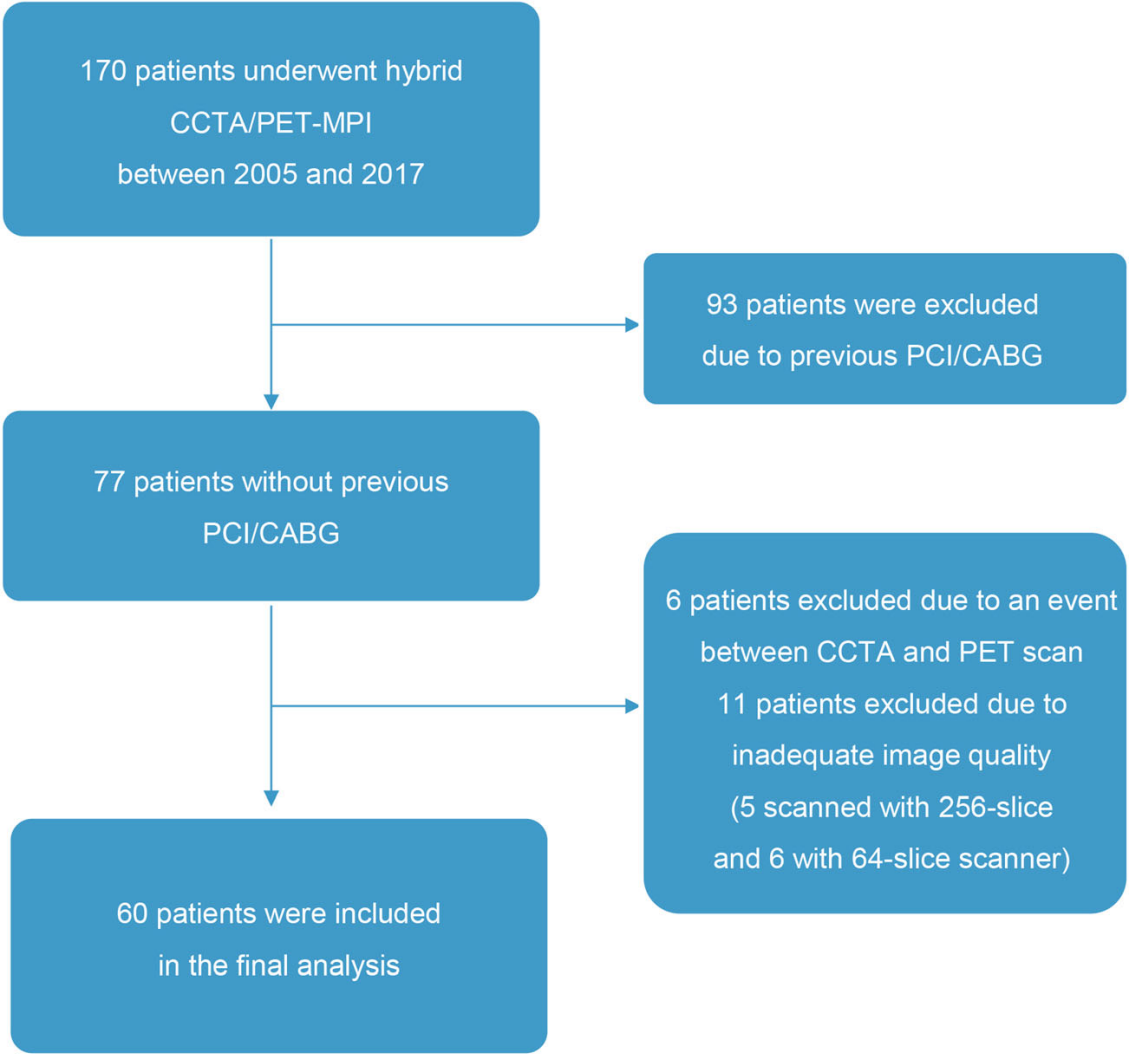
Study flow chart

Details on screening, exclusion, and eligibility for analysis of the study population are depicted in the study flow chart in Fig. 1. Sixty patients and 180 vessels were enrolled and analyzed. The mean heart rate during CCTA scans was 60.3 ± 11.1 bpm. CCTA and PET examinations were performed within 2 days (interquartile range 0–22). The baseline characteristics of the study population are summarized in Table 1.

**Table 1:** Baseline characteristics of the study population (*n* = 60)

Clinical characteristics	
Age (years)	64 ± 9.8
Gender (male)	43 (72)
BMI (kg/m^2^)	29 ± 6.6
Cardiovascular risk factors	
Arterial hypertension	31 (52)
Diabetes mellitus	6 (10)
Dyslipidemia	35 (58)
Positive family history	16 (27)
Smoking	26 (43)
Clinical symptoms	
Typical angina	14 (23)
Atypical angina	10 (17)
Unclear chest pain	12 (20)
Dyspnea	6 (10)
Asymptomatic	14 (23)
Other symptoms	4 (7)

Values given are absolute numbers and percentages (in brackets) or mean ± standard deviation. *BMI*, body mass index

### CCTA findings

CCTA confirmed > 50% stenosis and > 70% stenosis in at least one vessel in 44 (73.3%) and 15 (25%) patients, respectively. Vessel-wise, a stenosis > 50% was recorded in 78 vessels (43.3%), resulting in 19 (31.7%), 16 (26.7%), and 9 (11.7%) patients with 1-, 2- and 3-vessel disease, respectively. A stenosis > 70% was recorded in 22 vessels (12.2%). Mean LV mass and CAVi were 113.3 ± 37.4 g and 21.9 ± 8.5 mm^3^/g, respectively. ROC curve analysis of CAVi revealed an AUC of 0.711 (95% CI 0.561–0.861, *p* = 0.01) to predict ischemia. Based on Youden’s index, we identified an optimal cutoff value of 20.2 mm^3^/g. A CAVi equal or below this threshold was classified as low-CAVi (*n* = 24, 40%) and a value above this threshold was classified as high-CAVi (*n* = 36, 60%).

### Correlation of CAVi with ^13^N-ammonia PET measurements

Mean stress MBF, rest MBF, and MFR were lower in patients with low-CAVi, compared to patients with high-CAVi (1.66 ± 0.64 versus 2.32 ± 0.67 ml/min/g, 0.78 ± 0.17 versus 0.93 ± 0.32 ml/min/g and 2.09 ± 0.65 versus 2.56 ± 0.63 ml/min/g, respectively, *p* < 0.001 for all comparisons). The proportion of vessel territories with an abnormal stress MBF or an abnormal MFR was significantly higher in the low-CAVi group (59.7% versus 19% and 44.4% versus 18.1%, respectively, *p* < 0.001 for both comparisons). Interestingly, this was also true for vessel territories where CCTA did not show any stenosis > 50% (60% versus 14.3%, *p* < 0.001 with abnormal stress MBF and 40% versus 15.9% with abnormal MFR, *p* = 0.009) or any stenosis > 70% (57.1% versus 15.2%, *p* < 0.001 for abnormal MBF and 39.3% versus 16.2%, *p* = 0.001 for abnormal MFR).

**Fig. 2:**
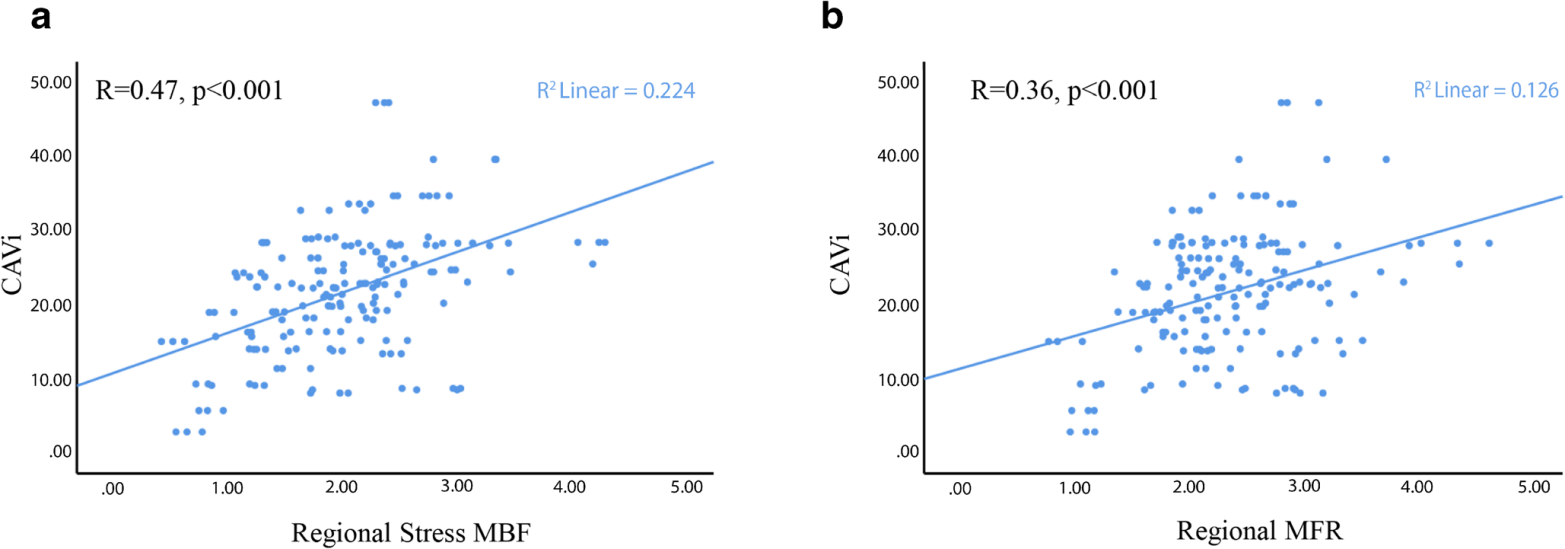
Scatterplots demonstrating the association between CAVi and regional stress MBF (panel **a**) and between CAVi and regional MFR (panel **b**)

There was a moderate correlation between CAVi and regional stress MBF and MFR (*R* = 0.47 and *R* = 0.36, respectively, *p* < 0.001 for both comparisons, Fig. 2)

**Fig. 3:**
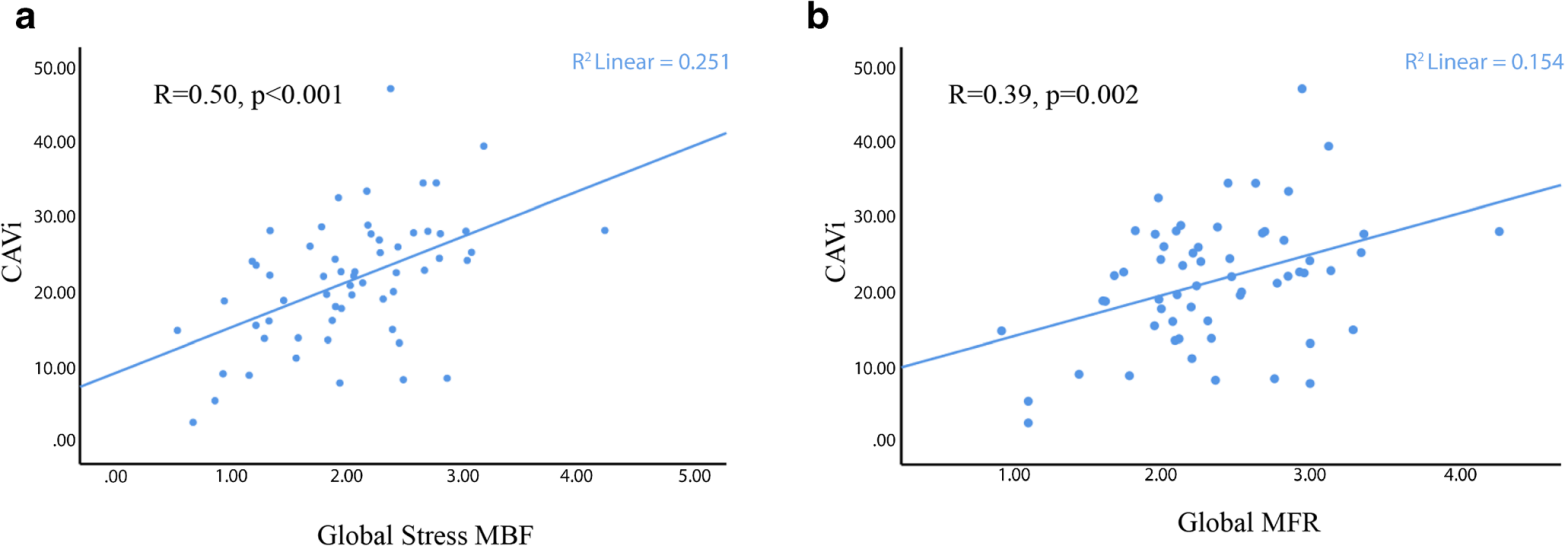
Scatterplots demonstrating the association between CAVi and global stress MBF (panel **a**) and between CAVi and global MFR (panel **b**)

There was a moderate correlation between CAVi and global stress MBF and MFR (*R* = 0.50, *p* < 0.001, and *R* = 0.39, *p* = 0.002, respectively, Fig. 3). Additionally, the proportion of patients with abnormal global MFR was higher in the low-CAVi group (33.3% versus 11.4%, *p* = 0.04). In the low-CAVi group (*n* = 24), 11 (45.8%) patients had abnormal stress MBF in all 3 vessel territories, 2 (8.3%) in 2 vessel territories, 6 (25%) in 1 territory, and 5 (20.8%) had normal stress MBF in all vessel territories. In contrast, in the high-CAVi group (*n* = 36), 4 (11.1%) patients showed abnormal stress MBF in all 3 vessel territories, 3 (8.3%) in 2 vessel territories, 2 (5.5%) in 1 territory, and 27 (75%) had normal stress MBF in all vessel territories (chi-square *p* < 0.001). Similar results were found for the analysis of MFR: As compared to the high-CAVi group, a larger proportion of patients in the low-CAVi group had abnormal MFR values in any of the 3 vessel territories, in 2 vessel territories, and in 1 vessel territory (25% versus 5.5%, 12.5% versus 8.33%, and 33.3% versus 19.44%, respectively, *p* = 0.03)

### Value of CAVi for abnormal stress MBF and ischemia prediction

A comparison of the ROC curves revealed that the AUC of CAVi for abnormal stress MBF prediction was larger compared to stenosis > 50% (*p* = 0.05) and similar with stenosis > 70% (*p* = 0.22). Moreover, the addition of CAVi increased significantly the AUC of the CCTA model, based on > 50% stenosis (*p* = 0.007).

**Fig. 4:**
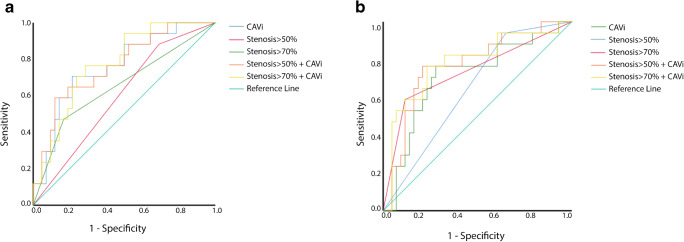
Areas under the curve of various models for abnormal stress MBF (panel **a**) and ischemia (panel **b**) prediction

Regarding ischemia prediction, there was no difference between the AUC of CAVi and the AUCs of the CCTA models (both stenosis > 50% and > 70%, *p* = 0.42 and *p* = 0.76, respectively). However, the addition of CAVi significantly increased the AUC of the CCTA model, based on > 50% stenosis (*p* = 0.03, Table 2 and Fig. 4).

**Table 2:** C-statistics of various models for abnormal stress MBF and ischemia prediction

	Abnormal stress MBF		Ischemia	
Model	C-statistic	95% CI	C-statistic	95% CI
1. Stenosis > 50%	0.596^†‡^	(0.460–0.722)	0.645*	0.561–0.861
2. Stenosis > 70%	0.652^#^	(0.517–0.771)	0.736	0.581–0.891
3. CAVi	0.758^‡^	(0.629–0.860)	0.711	0.561–0.861
4. Stenosis > 50% + CAVi	0.763^†^	(0.635–0.864)	0.770*	0.636–0.904
5. Stenosis > 70% + CAVi	0.775^#^	(0.647–0.873)	0.782	0.644–0.921

*CAVi*, coronary artery volume index; *CI*, confidence interval

^†^*p* value for comparison = 0.007

^‡^*p* value for comparison = 0.05

*p value for comparison = 0.03

^#^*p* value for comparison = 0.06

Table 3 summarizes the percentages of the patients correctly reclassified as having abnormal stress MBF or ischemia after the addition of CAVI on models based on stenosis severity (either > 50% or > 70%).

**Table 3:** Improvement in discrimination and risk reclassification for abnormal stress MBF and ischemia using CAVi in addition to stenosis severity

	IDI (95% CI)	Abnormal stress MBF		Normal stress MBF		NRI (95% CI)	IDI (95% CI)	Ischemia		Non-ischemia		NRI (95% CI)
		Risk up	Risk down	Risk up	Risk down			Risk up	Risk down	Risk up	Risk down	
Stenosis 50%	0.13 (0.03–0.23)	0%	19%	6%	33%	0.84 (0.33–1.35)	0.09 (0.03–0.15)	0%	0%	5%	14%	0.96 (0.47–1.45)
Stenosis 70%	0.07 (− 0.005–0.15	0%	19%	15%	36%	0.58 (0.04–1.12)	0.01 (− 0.003–0.03)	0%	0%	3%	0%	0.39 (− 0.15–0.93)

Baseline models included stenosis > 50%, age and gender or stenosis > 70%, age and gender. New model: baseline models + CAVi

In multivariate logistic regression analysis, CAVi was independently associated with abnormal stress MBF (odds ratio 0.90, 95% CI 0.82–0.998, *p* = 0.045) but not with ischemia. Results of the univariate and multivariate logistic regression analyses are given in Table 4.

**Table 4:** Univariate and multivariate regression analysis for prediction of abnormal stress MBF and ischemia as defined by ^13^N-ammonia PET

Predictors	Abnormal stress MBF				Ischemia					
	Univariate OR (95% CI)	*p* value	Multivariate OR (95% CI)	*p* value	Univariate OR (95% CI)	*p* value	Multivariate OR (95% CI)	*p* value	Multivariate OR (95% CI)	*p* value
Age	1.008 (0.95–1.07)	0.78			1.03 (0.97–1.1)	0.28				
Gender	9.85 (1.19–81.55)	0.03	6.62 (0.73–60.43)	0.09	4.02 (0.81–19.96)	0.09	1.91 (0.33–11.11)	0.47	2.83 (0.49–16.53)	0.25
≥ 3 Risk factors	2.51 (0.79–7.97)	0.12			0.92 (0.29–2.97)	0.89				
Symptoms	1.04 (0.72–1.49)	0.85			0.67 (0.44–1.02)	0.67				
Stenosis > 50%	3.36 (0.67–16.87)	0.14			8.57 (1.03–71.08)	0.047	5.58 (0.61–50.64)	0.13		
Stenosis > 70%	4.44 (1.27–15.53)	0.02	1.82 (0.42–7.96)	0.43	10.86 (2.84–41.57)	< 0.001			7.87 (1.72–36.15)	0.008
CAVi	0.87 (0.80–0.96)	0.003	0.90 (0.82–0.998)	0.045	0.91 (0.84–0.99)	0.02	0.93 (0.86–1.01)	0.08	0.98 (0.89–1.07)	0.58

**Fig. 5:**
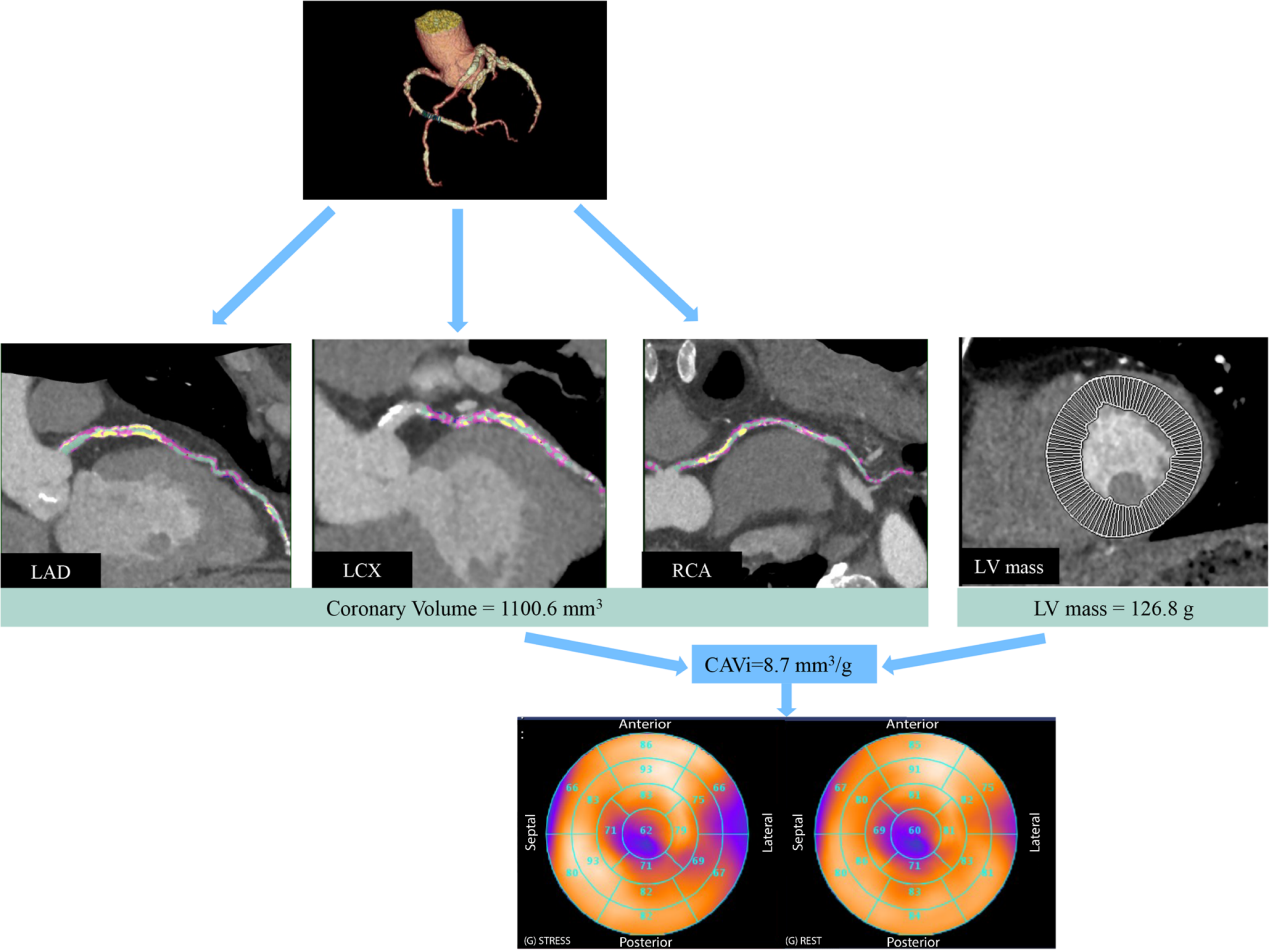
A 63-year-old male patient who underwent CCTA due to unstable angina. CCTA demonstrated obstructive CAD with high calcium burden and intermediate (i.e., 50–70%) stenosis in all three vessels. Global stress MBF as derived by ^13^N-ammonia PET was 2.88 ml/g/min. Coronary volume was calculated as 1100.6 mm3, and LV mass as 126.8 g, resulting in a CAVi of 8.7 mm^3^/g (i.e., low-CAVi). PET-MPI revealed ischemia in the left ventricular lateral wall

**Fig. 6:**
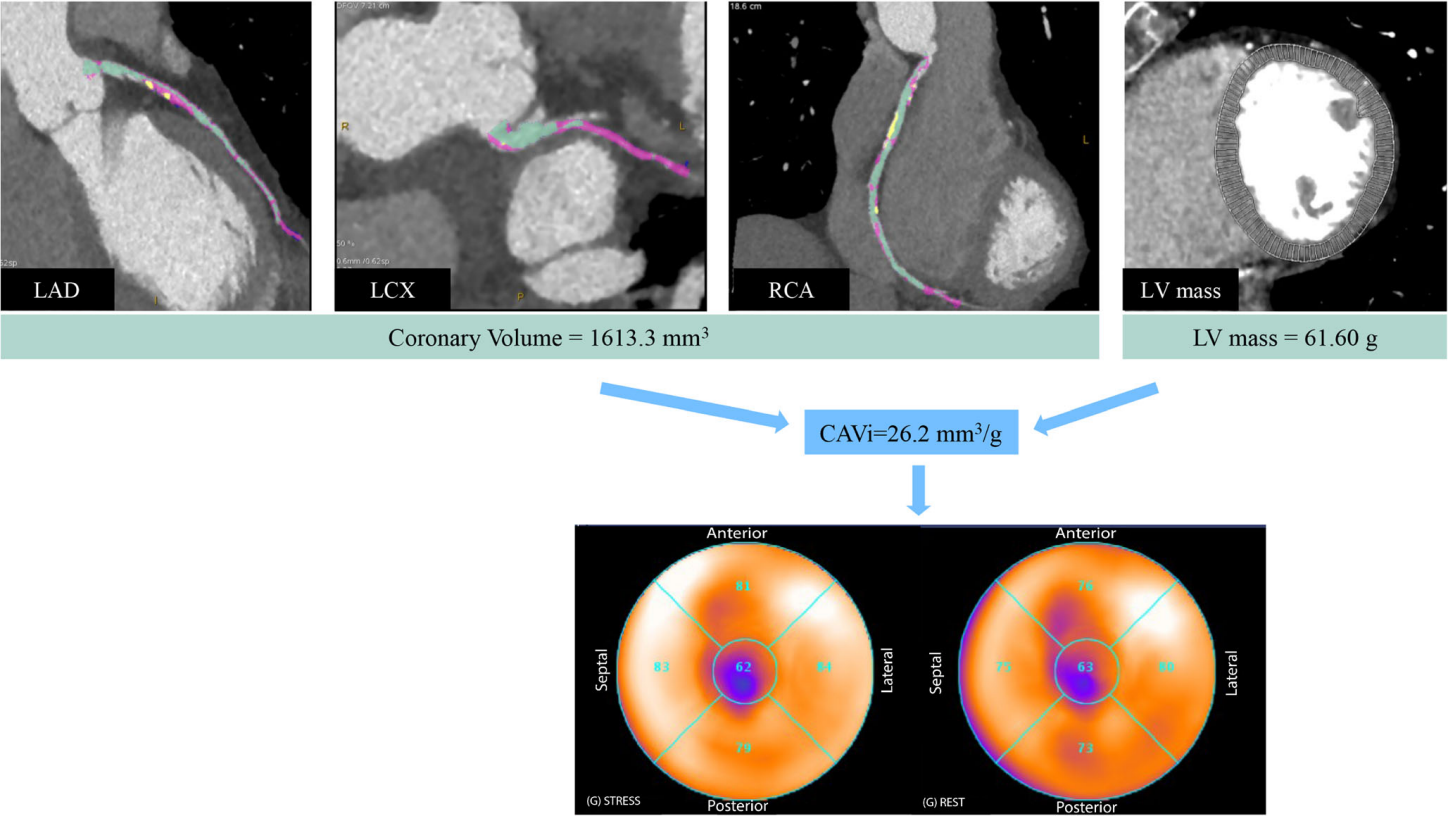
A 57-year-old female who underwent CCTA due to stable angina. CCTA demonstrated stenosis > 50% in LAD and LCx. Global stress MBF as derived by ^13^N-ammonia PET was 2.45 ml/g/min. Coronary volume was calculated as 1613.3 mm^3^ and LV mass as 61.60 g, resulting in a CAVI of 26.2 mm^3^/g (i.e., high-CAVi). PET-MPI revealed normal perfusion

 Figures 5 and 6 depict two representative cases, one with low-CAVi and one with high-CAVi, respectively, both with normal global stress MBF.

## Discussion

In the present study, we demonstrate that CAVi (a) correlates modestly with stress MBF and MFR, as derived from ^13^N-ammonia PET-MPI, both in vessel- and patient-based analyses; (b) it is independently associated with abnormal stress MBF; and (c) it exhibits incremental predictive value over stenosis severity for abnormal stress MBF and ischemia prediction.

There are several theories linking CAVi to myocardial perfusion. Firstly, according to allometric laws, there is a close linear relationship between coronary volume and left ventricular mass, verified in animal models [19]. Thus, it can be hypothesized that there is a critical threshold for coronary lumen volume per myocardial mass, below which adequate myocardial perfusion cannot be achieved, particularly in cases of diffuse—but not necessarily obstructive—coronary atherosclerosis. Indeed, De Bruyne et al showed an association between arteries with diffuse atherosclerosis but angiographically non-obstructive disease and abnormal FFR [5].

Further supporting this theory, in a recent study, Taylor et al evaluated for the first time the impact of CAVi on FFR values. The authors concluded that there is an association between low-CAVi and positive FFR values in invasive coronary angiography despite the absence of obstructive CAD [4]. In this study, a slightly lower CAVi cutoff value of 18.57 mm^3^/g, based on the median, was applied. However, as there are currently no standardized values for CAVi and because methods and study populations may vary, we used a cutoff based on ROC curve to predict ischemia.

A potential correlation of CAVi with qualitative myocardial flow parameters, derived non-invasively from PET-MPI (stress MBF and MFR), would further corroborate the validity of the basic concept for this theory, indicating CAVi as a surrogate marker of diffuse atherosclerosis. In a recent study by van Diemen et al [20], a very weak association was recorded, based on data derived from ^15^O-water PET-MPI. Interestingly, a cutoff of 20.7 mm^3^/g, very similar to the present study (20.2 mm^3^/g), was associated with both hyperemic blood flow and low invasive FFR. Furthermore, in accordance with our results, a higher ratio of vessel territories with abnormal stress MBF or MFR was observed in patients with low-CAVi. However, the correlations in our study, based for the first time on data derived from ^13^N-ammonia PET-MPI, for both stress MBF and MFR were stronger (*R* = 0.47, *p* < 0.001, and *R* = 0.36, *p* < 0.001 versus *R* = 0.148, *p* = 0.027, and *R* = 0.09, *p* = 0.14, respectively). Moreover, vessels without obstructive stenosis and either abnormal stress MBF or MFR were more common in patients with low-CAVi.

Impaired vessel responsiveness to endothelium-dependent vasodilatation due to diffuse intimal thickening is a second potential mechanism [4, 21]. Thus, a low-CAVi as derived from CCTA may be associated with impaired vasodilatory vessel response to nitroglycerin (which is applied before a CCTA scan) [22]. It may be hypothesized that CAVi, which is a marker obtained at the epicardial vessel-level, may be seen as surrogate marker for the entire coronary circulation, including the microcirculation. However, only a study assessing the intra-patient CAVi variability with and without nitrates could lend further support to the above theory.

The second important finding of the present study was the incremental value of CAVi in abnormal stress myocardial blood flow and ischemia prediction. In fact, the addition of CAVi significantly improved the predictive value of the stenosis-based model. This applies in particular when using the 50% stenosis threshold.

The discrepancy between the anatomic severity and the functional significance of a stenosis is well documented. CCTA provides a number of non-invasive parameters to further elucidate the potential hemodynamic relevance of a given lesion. These include transluminal attenuation gradient (TAG), vulnerable plaque characteristics, and CT-derived fractional flow reserve (FFR_CT_). Each technique tool has shown to feature specific strengths and limitations:

In brief, like CAVi, TAG is assessed solely based on CCTA-derived anatomy without the need of additional stress testing or image acquisitions. However, the validation of TAG against invasive FFR as the standard of reference has yielded conflicting results [14, 23, 24]. Morphological plaque characteristics indicative of vulnerability such as spotty calcifications, low attenuation areas, eccentric remodeling, or the napkin-ring sign have been shown to be associated with the presence of ischemia as derived from invasive FFR [25]. However, the absence of morphological vulnerability criteria does not exclude hemodynamical relevance of a given stenosis, limiting its negative predictive value. A further limitation arises from the dependence on very high image quality and spatial resolution [26]. Lastly, FFR_CT_ has been validated against invasive FFR in prospective studies, showing incremental diagnostic accuracy compared to CCTA alone [27]. On the other hand, the remote and time-consuming segmentation of vessel lumen and the dependence on very good image quality is currently limiting its clinical applicability.

The incremental value of CAVi to predict abnormal stress myocardial blood flow and ischemia suggests the potential clinical utility of this specific marker. Similarly to TAG and plaque vulnerability assessment, calculation of CAVi requires no stress testing nor additional scans and does not extensively depend on high spatial resolution, as do computational fluid dynamic calculations, for example, for CT-derived FFR. Moreover, the calculation can be performed on-site using commonly available software. Most importantly, the prognostic significance of CAVi has already been shown in a previous study, hinting at the potential for clinical application [3]. The validation, however, of exact cutoffs mandates large, prospective—and most importantly—multi-center and cross-vendor studies.

### Limitations

There are some limitations to the present study. Firstly, the retrospective nature of this study inherently introduced selection bias, and the results may have been affected by unmeasured confounders. Moreover, we did not apply the previously described segmentation for MFR (involving only segments distal to stenosis > 50%) [14]. Instead, we aimed to correlate the respective regional MFR of a vessel territory defined by the standard segmentation model to CAVi without, however, correcting for multiple measurements. Additionally, coronary lumen volumes were indexed to total myocardial mass, opting for a patient-phenotype predisposing for myocardial ischemia. A vessel-based segmentation of CAVi instead, relating a regional coronary artery lumen volume to the fraction of the myocardial mass subtended by the vessel territory, would require too many assumptions and may not be reliable [28]. Secondly, although CAVi assesses the vasodilatory response to nitroglycerin, it does not allow to directly assess the status of the microcirculation of the patient. Finally, as all CT scans were prospectively ECG-triggered and acquired at 75% of the RR-interval, the computed LV mass corresponds to the mid-diastolic rather than the true end-diastolic mass. However, mid-diastolic LV mass values have been validated and correlate well with the standard assessment of LV mass [29].

## Conclusions

CAVi is independently associated with abnormal stress myocardial blood flow. More importantly, CAVi exhibits incremental value to predict abnormal stress MBF and ischemia over findings from CCTA alone.

## Abbreviations

CAVi: Coronary artery volume indexed to left myocardial mass
CCTA: Coronary computed tomography angiography
MBF: Myocardial blood flow
MFR: Myocardial flow reserve
PET-MPI: Positron emission tomography myocardial perfusion imaging
